# Nivolumab drug holiday in patients treated for metastatic renal cell carcinoma: A real-world, single-centre experience

**DOI:** 10.3389/fonc.2022.960751

**Published:** 2022-08-12

**Authors:** Davide Bimbatti, Michele Dionese, Eleonora Lai, Nicolò Cavasin, Umberto Basso, Alvise Mattana, Francesco Pierantoni, Vittorina Zagonel, Marco Maruzzo

**Affiliations:** ^1^ Oncology 1 Unit, Istituto Oncologico Veneto, IOV – IRCCS, Padua, Italy; ^2^ Department of Surgical, Oncological and Gastroenterological Sciences, University of Padua, Padua, Italy; ^3^ Oncology 3 Unit, Istituto Oncologico Veneto, IOV – IRCCS, Padua, Italy

**Keywords:** mRCC, renal cell carcinoma, anti-PD1, immunotherapy, rechallenge

## Abstract

**Introduction:**

Immunotherapy with nivolumab (a monoclonal antibody that targets the programmed cell death protein 1, PD1) has become the standard treatment for patients with metastatic renal cell carcinoma (mRCC) after progression to single-agent tyrosine kinase inhibitors. However, the optimal duration of immunotherapy in this setting has not yet been established.

**Patients and methods:**

We retrospectively reviewed all patients treated with nivolumab at our institution from January 2014 to December 2021 and identified those who discontinued treatment for reasons other than disease progression (PD). We then associated progression-free survival (PFS) and overall survival following treatment cessation with baseline clinical data.

**Results:**

Fourteen patients were found to have discontinued treatment. Four patients (28.6%) ceased treatment due to G3/G4 toxicities, whereas the remaining ten (71.4%) opted to discontinue treatment in agreement with their referring clinicians. The median duration of the initial treatment with nivolumab was 21.7 months (7.5-37.3); during treatment, two patients (14.3%) achieved stable disease as the best response, and the remaining twelve (85.7%) a partial response. At a median follow-up time of 24.2 months after treatment discontinuation, 7 patients (50%) were still progression-free. The median PFS from the date of discontinuation was 19.8 months (15.2 - not reached); a radiological objective response according to RECIST and treatment duration of more than 12 months were associated with a longer PFS. Three patients were re-treated with Nivolumab after disease progression, all of whom achieved subsequent radiological stability.

**Conclusion:**

In our experience, the majority of patients who discontinued treatment in the absence of PD were still progression-free more than 18 months after discontinuation. Patients whose initial treatment duration was less than 12 months or who did not achieve a radiological objective response had a greater risk of progression. Immunotherapy rechallenge is safe and seems capable of achieving disease control.

## Introduction

Renal cell carcinoma (RCC) is the most common type of kidney cancer in adults and accounts for 3-5% of new cancer diagnoses each year (1, 2). Nowadays, incidental early-stage RCC diagnoses account for the majority of new cases, but a significant proportion of patients with localised disease will still develop metastases at some point in time (3).

In recent years, immunotherapy in the form of immune checkpoint inhibitors (ICIs) has revolutionised the treatment of metastatic RCC.

Nivolumab, an ICI that targets the programmed cell-death protein 1 (PD1), has become the standard treatment for patients with mRCC following progression to single-agent tyrosine kinase inhibitors (TKI) (4). In combination with cabozantinib (a TKI) or ipilimumab (an ICI that targets the anti-Cytotoxic T-Lymphocyte Antigen 4), it is considered to be one of the standard treatments in previously untreated patients (5–7).

However, the maximum duration of treatment differed in those trials. In the 2015 Checkmate 025 trial (nivolumab vs. everolimus for pre-treated mRCC), the first trial that paved the way for nivolumab in the management of RCC, treatment continued until disease progression or the development of treatment-limiting toxicities (4). In the 2018 Checkmate 214 trial (nivolumab plus ipilimumab as first-line treatment), treatment with nivolumab was initially planned to continue until disease progression or the development of toxicities, but a subsequent amendment allowed the patient to discontinue therapy after two years (5, 8). Finally, in the 2021 Checkmate 9ER trial (nivolumab plus cabozantinib as first-line therapy), treatment with nivolumab had a maximum duration of two years from the start of treatment (6).

The reason for limiting the maximum duration of immunotherapy treatment is the growing body of evidence indicating that the disease’s clinical control is often long-lasting and may be maintained even after therapy is discontinued. In fact, due to their unique mechanism of action, ICIs are capable of achieving long-term disease control in many solid malignancies, even after treatment discontinuation or interruption (9–12). Therefore, prolonged and ongoing treatment may not always be necessary for all patients.

Data from retrospective analyses indicated that treatment interruption after a certain number of cycles could be safe for selected patients (11–13). Moreover, other studies demonstrate the feasibility of presenting a rechallenge with ICIs in the event of disease progression following prior immunotherapy (14, 15).

A patient-tailored “stop and go” approach could be an alternative option for selected patients in order to reduce overtreatment, limit the occurrence of treatment-related toxicities, and improve the possible financial toxicity of those therapies without compromising the treatment’s oncological results (in terms of clinical benefit and preservation of quality of life).

This paper presents a retrospective analysis of patients treated with nivolumab at our institution, who opted to discontinue treatment in the absence of disease progression.

## Patients and methods.

We retrospectively reviewed all patients treated with nivolumab at our institution from January 2014 to December 2021 and identified those who discontinued treatment for reasons other than disease progression. Clinical data were extracted from electronic patient records.

Inclusion criteria included a histological diagnosis of RCC, previous treatment with nivolumab interrupted in the absence of PD, and the availability of all necessary data.

From electronic patient charts, we collected baseline clinical data, the reason for treatment discontinuation, the treatment’s oncological outcome (including duration of initial treatment, best radiological response, development of immune-related toxicities, date of disease progression, and date of death or last follow-up), and data about subsequent treatments administered after disease progression. Adverse events were graded in accordance with the Common Terminology Criteria for Adverse Events (CTCAE) v5.0; radiological response was defined using Response Evaluation Criteria in Solid Tumors (RECIST) v1.1 criteria.

Treatment duration was defined as the time between the first and last dose of nivolumab. Progression-free survival (PFS) was calculated using the Kaplan-Meier method from the date of treatment interruption to the date of disease progression or death (whichever occurred first); progression-free survival was censored at the last patient follow-up visit without progression. Overall survival was calculated from the date of drug interruption to the date of death from any cause. For patients re-treated with nivolumab after disease progression, PFS for the second course of immunotherapy was calculated from the beginning of the second course until the occurrence of new disease progression.

Key metrics were summarised by means of descriptive statistics. Patient PFS and OS were compared using the log-rank test and Cox’s proportional hazards method (when applicable). We performed univariate and multivariate analyses to determine the association between baseline characteristics and PFS from the time of treatment discontinuation; the covariates that showed any association with the oncological outcome with a p value of at least less than 0.1 in the univariate analyses were included in the multivariate analysis. Results were classified as statistically significant if their p-values were < 0.05. All statistical analyses were performed with “R” v4.0.5 and the “survival” package v2.44-1.1.

At the time of their first visit to our institution, all patients gave their written consent for the use of their clinical data for scientific purposes. The study was conducted in accordance with the Declaration of Helsinki. Data collection was approved by the local Ethical Committee.

## Results

### Patient characteristics

Fourteen patients were found to have discontinued treatment for reasons other than disease progression. The median age was 77.7 years (range: 42.3-82.1 years). Eleven patients had been diagnosed with clear cell RCC (78.6%), one with papillary RCC, one with chromophobe RCC and one with RCC not otherwise specified. Twelve patients were treated with nivolumab in the second-line setting, while two patients were treated in the third-line. All but one patient had received nephrectomy prior to treatment. All patients were in good clinical condition at the start of Nivolumab treatment (ECOG PS of 0 or 1); 5 patients were classified as belonging to the good risk class according to IMDC criteria, while the remaining 9 patients were classified in the intermediate risk class; none of the patients were considered to be at poor risk. Patient clinical characteristics are summarised in **Table 1**.

**Table 1:** Patient clinical characteristics.

Characteristics	Number of patients
**Gender**	
Male Female	10 (71.4%) 4 (28.6%)
**Age (years)**	
Mean (range) >70 years (%)	77.6 (42.3-82.1) 12 (85.7%)
**Histology**	
Clear Cell Other histologies	11 (78.6%) 3 (21.4%)
**Previous nephrectomy**	
Yes No	13 (92.9%) 1 (7.1%)
**Metastases locations (number of patients)**	
Lymph nodes Bone Liver Lung Small tissue Adrenal Others	7 (50%) 3 (21.4%) 3 (21.4%) 10 (71.4%) 3 (21.4%) 2 (14.3%) 3 (21.4%)
**Performance Status (ECOG)**	
0 1	4 (28.6%) 10 (71.4%)
**IMDC risk classification**	
Good Intermediate Poor	5 (35.7%) 9 (64.3%) 0 (0%)
**Setting**	
II line III line	12 (85.7%) 2 (14.3%)

### Initial treatment details

The median duration of initial treatment with nivolumab was 21.7 months (7.5-37.3). During treatment, two patients (14.3%) achieved stable disease as the best radiological response, while the remaining twelve patients (85.7%) achieved a partial response. Twelve patients (85.7%) developed immune-related adverse events of any grade during therapy, requiring at least a brief interruption of nivolumab or treatment with systemic corticosteroids; four patients reported the onset of grade 3/4 toxicities (one grade 3 colitis, two grade 3 myocarditis and three grade 3 hypertransaminasemia). Data on treatment outcomes are reported in **Table 2**.

**Table 2:** Details of patient baseline characteristics, initial treatment, therapeutic pause and post-progression course.

	Histology	Age (y)	PS	Tx duration (m)	BR	Reason for interruption	iRAE during initial tx	Drug holiday (m)	PD	Tx at PD	BR at rechallenge	Duration of rechallenge
1	RCC NOS	80.2	0	8.9	PR	Decision	Skin reaction (G2)	5.1	No	NA	NA	NA
2	Clear cell RCC	81	1	27.8	SD	Decision	None	15.2	Yes	Nivolumab	SD	12
3	Clear cell RCC	82.1	0	24.7	PR	Decision	None	26.3	No	NA	NA	NA
4	Clear cell RCC	80	1	24.3	PR	Decision	Skin reaction (G2)	16.1	Yes	SBRT	NA	NA
5	Clear cell RCC	75.9	0	21.4	PR	Decision	Uveitis (G2)	25	No	NA	NA	NA
6	Clear cell RCC	82	1	37.3	PR	Decision	Skin reaction (G2)	19.8	Yes	BSC	NA	NA
7	Clear cell RCC	53	0	12.9	PR	Decision	Arthralgia (G2)	18.4	No	NA	NA	NA
8	Clear cell RCC	74.6	0	22	PR	Decision	Pneumonia (G2)	37.1	No	NA	NA	NA
9	Papillary RCC	78.7	1	7.5	PR	irAE	Hypertransaminasemia (G3), hyperglycaemia/diabetes (G2)	10	Yes	Nivolumab	SD	5
10	Clear cell RCC	77.6	1	22.7	PR	Decision	Skin reaction (G2)	35.7	No	NA	NA	NA
11	Clear cell RCC	42.3	0	8.7	SD	irAE	Hypertransaminasemia (G3)	5.6	Yes	Nivolumab	SD	4
12	Chromophobe RCC	74.3	1	32.7	PR	irAE	Colitis (G3)	13.5	Yes	Cabozantinib	PR	NA
13	Clear cell RCC	73	1	7.9	PR	irAE	Myocarditis (G3)	15.6	Yes	BSC	NA	NA
14	Clear cell RCC	77.8	1	11.2	PR	Decision	Skin reaction (G2)	5.7	No	NA	NA	NA

RCC, renal cell carcinoma; NOS, not otherwise specified; BR, best response; PR, partial response; SD, stable disease; PS, performance status (sec ECOG); Tx, treatment; BSC, best supportive care; iRAE, immuno-related adverse event; G2 or G3, grading per CTCAE; SBRT, sterotactic body radiation therapy; NA, Not Applicable.

### Cause of discontinuation, PFS from interruption and factors associated with PFS

Ten patients (71.4%) opted to discontinue treatment in agreement with their referring clinicians; however, for 5 of these patients (50%), the previous occurrence of low-grade (G1-G2) adverse events was an important factor in their decision. The other four patients (28.6%) discontinued treatment after developing G3/G4 toxicities.

At a median follow-up time of 24.2 months after treatment discontinuation, 7 patients (50%) were still progression-free. For 5 of the 7 patients who progressed, radiological progression was defined by the enlargement of known pre-existing lesions, and for the other 2, by the emergence of metastases at new sites (two new lesions in the liver and a brain metastasis, respectively).

**Figure 1:**
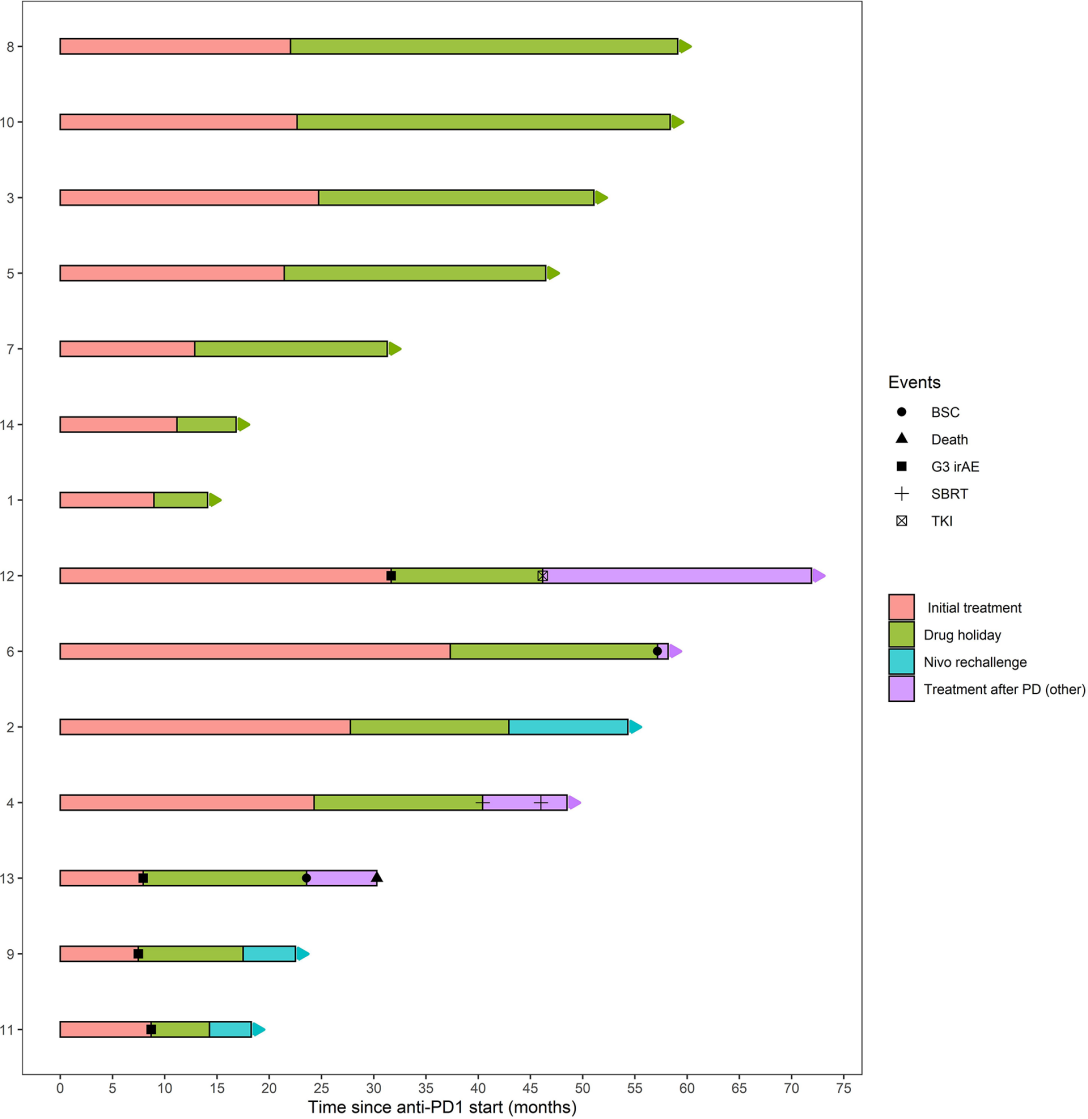
Duration of initial treatment, treatment free interval and subsequent therapies in patients with (below) or without (above) disease progression after nivolumab interruption. BSC, best supportive care; SBRT, stereotactic body radiation therapy.

The median PFS from the date of discontinuation until disease progression was 19.8 months (15.2 - not reached); the median overall survival was not reached, with just one patient having died by the time of data cut off. Data on the post-interruption outcomes are reported in **Table 2**; **Figure 1**.

At univariate analysis, stable disease as the best radiological response and a treatment duration of less than 12 months were associated with a worse PFS (**Table 3**); sex, IMDC risk group, performance status at the start of the interruption and the development of immune-related adverse events (irAEs) during treatment were not significantly associated with PFS (**Table 3**). The prognostic value of treatment duration and radiological response were maintained at multivariate analysis (**Table 3**).

**Table 3:** Univariate and multivariate analyses of characteristics associated with PFS after nivolumab discontinuation.

Characteristics	Univariate HR (95% CI)	p	Multivariate HR (95% CI)	p
**Gender**				
M vs. F	1.41 (0.27-7.4)	0.68		
**Age (years)**	0.97 (0.91 – 1)	0.46		
**Duration of initial treatment**				
> 12 vs. ≤ 12 months	0.12 (0.019-0.73)	**0.02**	0.06 (0.01-0.62)	**0.018**
**IMDC risk group**				
Interm. vs. Good	0.61 (0.13-2.8)	0.52		
**Occurence of irAEs**				
Yes vs. No	1.3 (0.15 - 11)	0.82		
**Basal PS**				
1 vs. 0	0.82 (0.16-4.3)	0.82		
**Radiological BR**				
SD vs. PR	7.9 (1.1-57)	**0.04**	18.1 (1.38-237)	**0.028**

HR, hazard ratio; M, male; F, female; BR, best response; PR, partial response; SD, stable disease; PS, performance status (sec ECOG); iRAE, immuno-related adverse event; IMDC, International Metastastic RCC Database Consortium.Bold values are statistically significant values.

### Treatment after disease progression

After disease progression, two patients were considered ineligible for other oncological treatments due to their poor clinical condition and were, therefore, only treated with best supportive care. One patient, whose CT scan revealed an oligoprogressive disease, was successfully treated twice in succession with stereotactic ablative radiotherapy and has not yet begun additional systemic therapy.

Systemic therapy was initiated for the other four patients: due to the previous occurrence of immune-related colitis, one patient started third-line treatment with cabozantinib; the other three patients were re-treated with nivolumab. For two of these three patients, the cause for initial discontinuation was the emergence of an irAE (two grade 3 hypertransaminasemia) At the time of data cut off, the patients re-treated with nivolumab had been treated for 4, 5 and 12 months and are all progression-free; to date, no immune-related adverse event of any grade has been reported for either of them. Data on the treatments administered after disease progression and outcomes are reported in **Table 2**; **Figure 1**.

## Discussion

Immunotherapy has drastically improved the prognosis and natural history of patients with advanced renal cell carcinoma. The role of immunotherapy has been enhanced with the publication of recent trials, and a combination treatment with an immune checkpoint inhibitor is currently considered to be the standard of care in the first-line setting (7). Nevertheless, the definition of the optimal immunotherapy treatment duration is a clinical need that is still unmet.

Despite the fact that in the first and older trials, treatment with immune checkpoint inhibitors was continued until disease progression or the development of severe toxicities, ICIs have been shown to achieve long-term disease control even in the event of interruption (for example, due to adverse events or decisions by physicians or patients) (11, 12). There is strong biological evidence to support the fact that for many patients, especially those who are able to achieve a dimensional response at the radiological assessment, the continuation of treatment until progression occurs is not always necessary (13).

Long-term follow-up analysis of clinical trials using ICIs in melanoma and NSCLC demonstrated that many patients maintain the therapeutic benefit long after the end of treatment (11, 12).

In the case of RCC, 27 patients with a response to nivolumab discontinued treatment in the Checkmate 025 trial and never received additional subsequent systemic therapy (with a median treatment-free interval of 12.7 months); 13 of these patients were still alive and free from disease progression at the last follow-up (10).

Many clinical trials are currently set for a maximum 2-year period of ICI treatment for all patients enrolled (6, 16). However, it is not clear whether this fixed duration is totally necessary or whether treatment could be discontinued earlier in selected patients (or should be continued for other patients, even after this arbitrary cut off).

In metastatic melanoma, a retrospective analysis of patients treated with anti-PD1 (pembrolizumab or nivolumab) for a median initial treatment duration of 12 months showed that the risk of relapse after treatment discontinuation was low, particularly in patients who achieved complete radiological response during treatment (13).

Conversely, in non-small cell lung cancer (NSCLC), a randomised trial revealed that a fixed duration of one year seems to be inferior, in terms of PFS and OS, to continuous treatment with nivolumab in the whole population (17).

Several authors have investigated the optimal duration and management of ICI treatment for RCC. In a recent phase II trial, 5 out of 12 patients (42%) who opted to discontinue nivolumab after achieving a radiological response within the first 6 months of treatment were progression-free one year after the discontinuation of treatment (18).

Ornstein et al. conducted a phase II trial to evaluate the outcomes of intermittent treatment with nivolumab in a similar setting; of five patients who opted to discontinue nivolumab after obtaining a radiological reduction of 10% in tumor size, only one patient had to restart treatment at a median follow-up of 48 weeks (19).

These small trials demonstrate that, for some patients, treatment interruption could be a viable option, but additional and larger studies are needed to increase the level of evidence and refine patient selection.

However, following the decision to discontinue treatment, another important unanswered question concerns the immunotherapy rechallenge’s efficacy. Retrospective analysis in patients with other solid malignancies revealed an interesting response rate and a clinical benefit in patients re-treated with immunotherapy after disease progression (with the same ICI after a therapeutic pause or with a different ICI in the event of PD during treatment) (15, 20).

In a retrospective, multicentric analysis of renal cell carcinoma, Ravi et al. found a response rate of 23% with low incidence of severe adverse events in a cohort of 69 patients (50 of them discontinued initial treatment due to PD and 16 due to irAEs) who underwent anti-PD1/anti-PDL1 rechallenge treatment (14). The occurrence of grade 3-4 irAEs was reported by 18 patients (26%) during the first immunotherapy course and by 11 patients during the rechallenge, but only 3 of these patients had previous G3 toxicity during initial treatment (14).

The rechallenge strategy must be evaluated differently depending on whether the decision to discontinue therapy was due to the occurrence of toxicities or due to the patients’ or physicians’ preferences, as opposed to the progression of disease during treatment. Unfortunately, many studies, such as the abovementioned ones, did not distinguish between patients whose disease was under control or progressing when they discontinued treatment. These clinical situations are clearly distinct, and the results of re-treatment in one setting may not be applicable in another.

In fact, recent trials specifically designed for patients after progression or a lack of response to treatment with a single ICI are evaluating the intensification strategy using combination treatment (TKI plus anti-PD1 or anti-PD1 plus another ICI) rather than a single ICI (21, 22).

The final important question concerns the rechallenge’s toxicity profile. Many retrospective analyses demonstrated that, for patients who previously discontinued immunotherapy due to the occurrence of irAEs, these irAEs do not typically recur after the immunotherapy rechallenge’s commencement. Moreover, irAEs are usually milder and more manageable during rechallenge (15, 23–25). Due to the retrospective nature of these studies, toxicity profile data must be interpreted with caution. In fact, selection bias is a significant limitation, and it is likely that the patients selected for a rechallenge were those who only experienced non-life-threatening, minor and transient adverse events (AEs) in the first course of therapy.

The majority of patients in our population who opted to discontinue treatment were safe and progression-free after more than one year from the start of the therapeutic break. As reported by other authors, the risk of progression was lower in patients who had been treated for more than 12 months and in patients who had previously achieved an objective radiological response. Re-treatment appeared to be safe for patients who had progressed; it is interesting to note that, despite the limitations of a short follow-up, no treatment-related adverse events were reported, in spite of the fact that two of the patients had initially discontinued treatment due to grade 3 toxicities (hypertransaminasemia). Accordingly, we decided not to re-treat the patient who had previously reported grade 3 colitis.

Our analysis has several limitations. Due to the retrospective design, there was a selection bias in the population, which consisted of patients with a very good clinical condition and good prognostic characteristics at baseline. The small sample size limited the possibility of finding prognostic and predictive indicators for a prolonged drug holiday period; this could explain why many well-established prognostic factors, such as the IMDC class and performance status, did not seem to be associated with this PFS. Finally, radiological evaluation was performed as per the clinician’s decision, and radiological images were not re-examined.

## Conclusion

In our experience, the discontinuation of nivolumab treatment in a cohort of highly selected patients seems to be safe and capable of sustaining the disease’s long-term clinical control. Treatment duration of more than one year and the achievement of a radiological objective response were prognostic of longer progression-free survival from the date of treatment discontinuation. Rechallenge with nivolumab after the occurrence of progression seemed to be safe for the selected patients, including those patients who had previously reported the occurrence of certain toxicities.

More studies are urgently needed to determine the optimal duration and management of treatment with ICIs, especially given the ever increasing importance of immunotherapy. An improvement in the selection of patients who can safely discontinue treatment with ICIs could result in a dramatic improvement in treatment customisation and individualisation.

## Data availability statement

The data analyzed in this study is subject to the following licenses/restrictions: Available upon request. Requests to access these datasets should be directed to marco.maruzzo@iov.veneto.it.

## Ethics statement

The studies involving human participants were reviewed and approved by Comitato Etico IOV IRCCS. The patients/participants provided their written informed consent to participate in this study.

## Author contributions

MM, DB, and VZ study design. EL, NC and AM data collection. MD, DB and FP data analysis. MD, DB and MM data interpretation and review. UB, DB and MM supervision. All authors: final review and approval of the final paper.

## Funding

This research received “Ricerca Corrente” funding from the Italian Ministry of Health to cover publication costs.

## Conflict of interest

The authors declare that the research was conducted in the absence of any commercial or financial relationships that could be construed as a potential conflict of interest.

## Publisher’s note

All claims expressed in this article are solely those of the authors and do not necessarily represent those of their affiliated organizations, or those of the publisher, the editors and the reviewers. Any product that may be evaluated in this article, or claim that may be made by its manufacturer, is not guaranteed or endorsed by the publisher.
